# CuInS_2_/Mg(OH)_2_ Nanosheets for the Enhanced Visible-Light Photocatalytic Degradation of Tetracycline

**DOI:** 10.3390/nano9111567

**Published:** 2019-11-05

**Authors:** Xiaogang Zheng, Yiting Mao, Jing Wen, Xiaojin Fu, Xinhui Liu

**Affiliations:** 1 College of Chemistry and Chemical Engineering, Neijiang Normal University, Neijiang 641100, Chinafuxian2005@yahoo.com.cn (X.F.); 2 Key Laboratory of Comprehensive and Highly Efficient Utilization of Salt Lake Resources, Key Laboratory of Salt Lake Resources Chemistry of Qinghai Province, Qinghai Institute of Salt Lakes, Chinese Academy of Sciences, Xining 810008, Qinghai, China; 3 State Key Laboratory of Water Environment Simulation, School of Environment, Beijing Normal University, Beijing 100875, China

**Keywords:** CuInS_2_/Mg(OH)_2_ nanosheets, heterojunction, tetracycline hydrochloride, photocatalysis

## Abstract

CuInS_2_/Mg(OH)_2_ (CIS/Mg(OH)_2_) nanosheets have been prepared for the visible light activated photodegradation of tetracycline hydrochloride (TCH). The introduction of CuInS_2_ has proven to enhance the photocatalytic activity of Mg(OH)_2_ nanosheets. It’s ascribed to the enhanced transfer and separation of charge carriers at the junction interface between CuInS_2_ and Mg(OH)_2_. The photocatalytic activity of obtained CIS/Mg(OH)_2_ is greatly affected by CuInS_2_ content, pH value, and inorganic ions. Among these samples, 2-CIS/Mg(OH)_2_ exhibits the excellent photocatalytic activity and durability for the visible light driven removal of TCH after five cycle times. Atomic force microscope (AFM) images indicate that the surface roughness of 2-CIS/Mg(OH)_2_ is intensively influenced in adsorption-photocatalysis process. The •O_2_^−^ and •OH radicals are vital for the visible light driven photocatalytic activity of 2-CIS/Mg(OH)_2_ for TCH removal.

## 1. Introduction

Tetracycline hydrochloride (TCH, C_22_H_24_N_2_O_8_·HCl), as a broad-spectrum antibiotic, has been intensively applied for the therapy of human and livestock infections [1]. Due to the serious abuse and ineffective metabolization of TCH by the human body and livestock, its residue has become one of the largest source producers of non-biodegradable pollution damaging the human health and ecosystems [2,3]. Many strategies such as adsorption [4,5,6], biodegradation [7,8], coagulation [9], flotation [10], and advanced oxidation [11,12,13,14] are thus developed for the removal of TCH to reach a permissible level of ecological environment. As a typical advanced oxidation route, semiconductor-based photocatalysis has been paid great attention due to its low cost, non-pollution, and high efficiency [15,16,17,18,19,20]. In such a sustainable and green process, a proper semiconductor serves as the photocatalyst to participate in the oxidation reaction of TCH removal under solar light irradiation.

As a direct I-III-VI band gap semiconductor consisting of one divalent, one trivalent, and two divalent cations, copper indium sulfide (CuInS_2_) is considered as a potential visible-light photocatalyst owing to its narrow band gap (~1.5 eV), large optical absorption coefficient (α > 10^5^ cm^−1^) and efficient solar energy conversion [21,22,23]. The phase structures such as wurtzite, zinc blende and chalcopyrite of CuInS_2_ can greatly affect its luminescence property. For example, hexagonal CuInS_2_ with wurtzite structure is designed to enhance light-absorbing and to increase charge transfer because of the anisotropic crystal structure [24]. In addition, the abundant surface defects serve as the non-radiative recombination sites of charge carriers, accelerating the photocatalytic reaction on the surface of CuInS_2_ [25]. Unfortunately, CuInS_2_ suffers from the poor durability because of the photo-corrosion and surface oxidation under the long-term irradiation [26]. In fact, the acute photo-corrosion is ascribed to the excess holes assembled in the valance band of CuInS_2_. Therefore, the key issue of the enhanced stability and recyclability is to transfer the photoexcited holes from the valence band of CuInS_2_. Except for the hole-capture carrier and the sacrificial agents for the suppression of photoinduced corrosion, the heterojunction combined with at least two materials as well as has been confirmed to accelerate the hole transfer and enhance the stability [27,28,29]. For example, CuInS_2_/ZnS-TiO_2_ [30], CuInS_2_/g-C_3_N_4_ [31], CuInS_2_/Bi_2_WO_6_ [32], rGO/CuInS_2_ [33], and CuInS_2_/ZnS [34] have proven to expose the sufficient active sites and promote the transfer and separation of charge carriers at the junction interface. The efficient hole-capture materials are still explored for improving the hole transfer and affording the stability of CuInS_2_.

Magnesium hydroxide (Mg(OH)_2_), as a typical two-dimensional material with a type-II band structure, has been widely applied in photovoltaic devices and solid-state electronics owing to its rapid electron transport, low exciton recombination rate and efficient photon-harvesting capacity [35,36,37,38,39,40,41,42,43,44]. In contrast with the unstable or metastable two-dimensional materials such as black phosphorus, GaTe and MoTe_2_, the passivated surfaces created by O–H bonds composing of oxygen and hydrogen atoms are beneficial for the prolonged environmental stability of Mg(OH)_2_ [36]. To further extend the application of Mg(OH)_2_ in photocatalytic filed, Mg(OH)_2_-based heterojunctions such as Mg(OH)_2_/WS_2_ [40, 41], Mg(OH)_2_/MoS_2_ [42], and Mg(OH)_2_/AlN [43] have been designed for the extraordinary optical and electronic properties. Mg(OH)_2_ based heterostructure can constantly separate the photoexcited electron-hole pairs and owns the decent band edge positions for photocatalytic redox reaction in the built-in electric filed [45,46,47]. It’s appealing and desirable to acquire Mg(OH)_2_-based heterojunctions for the visible light driven environmental remediation. To our best knowledge, there are no previous works about the enhanced photocatalytic activity of Mg(OH)_2_ nanosheets coupled with CuInS_2_.

In this work, CuInS_2_-docacted Mg(OH)_2_ (CIS/Mg(OH)_2_) nanosheets were developed for the visible light activated photocatalytic degradation of TCH. This heterojunction can modulate the interactions between Mg(OH)_2_ nanosheets and CuInS_2_ nanoparticles for the enhanced photocatalytic activity in visible region. CIS/Mg(OH)_2_ heterojunction is expected to present better photocatalytic capacity than Mg(OH)_2_ alone for the TCH removal.

## 2. Materials and Methods 

### 2.1. Preparation of Catalysts

Mg(OH)_2_ nanosheets were prepared via a hydrothermal approach. Briefly, 3 mmol magnesium nitrate (Mg(NO_3_)_2_·6H_2_O) and 1.0 g polyvinyl pyrrolidone (PVP, M = 58,000) were dispersed into 50 mL deionized water and then intensively stirred at room temperature for 2.0 h. Subsequently, ammonia solution (NH_4_OH, 25 wt. %) with a stoichiometric ratio of Mg^2+^/OH^−^ (1:2) was slowly added into the above solution with an intensive stirring at room temperature for 3.0 h. The mixture solution was further transferred into a 100 mL Teflon-lined autoclave with the continuous heating at 423 K for 5.0 h. After cooling down to room temperature, the suspension was centrifuged, washed with deionized water for three times, and dried at 353 K for 5.0 h to obtain Mg(OH)_2_ nanosheets.

CuInS_2_/Mg(OH)_2_ composites were also prepared by a hydrothermal route. Typically, 0.1 g obtained Mg(OH)_2_ bulks was dispersed into a 40 mL deionized water, and then cuprous chloride (CuCl), indium trichloride (InCl_3_·4H_2_O) and thioacetamide (CH_3_CSNH_2_) with their stoichiometric proportion were added into the above solution to achieve different loading content of CuInS_2_ (3, 5, 7, and 9 wt. %). After stirred at room temperature for 2.0 h, this suspension was placed at a 50 mL Teflon-lined autoclave and heated at 433 K for 12.0 h. When cooled down to room temperature, the above suspension was centrifuged, washed, and dried at 333 K for 10.0 h to obtain CuInS_2_/Mg(OH)_2_ with the CuInS_2_ content of 3, 5, 7 and 9 wt. %, which were respectively named as 1-CIS/Mg(OH)_2_, 2-CIS/Mg(OH)_2_, 3-CIS/Mg(OH)_2_, and 4-CIS/Mg(OH)_2_.

### 2.2. Characterization of Catalysts

CIS/Mg(OH)_2_ composites were analyzed examined by X-ray diffraction (XRD, Bruker D8), field emission scanning electron microscopy (FE-SEM, Hitachi S-3400), transmission electron microscopy (TEM, FEI Tecnai G2 F20), X-ray photoelectron spectroscopy (XPS, Escalab 250), atomic force microscope (AFM, NT-MDT model BL222RNTE), N_2_ adsorption-desorption curves (NOVA-2020), inductively coupled plasma optical emission spectrometer (ICP-OES, Varian 710-ES), UV–Vis diffuse reflectance spectra (UV-Vis DRS, Hitachi U-4100) and Photoluminescence (PL, FLSP 920). The photoelectrochemical behaviors of these samples were investigated by a photoelectric instrument (CEL-PECX2000, Beijing CEL Tech. Co., Ltd., China) equipped with a Vertex. C. EIS (electrochemical impedance spectroscopy) electrochemistry workstation (Ivium Technologies B.V., Holland) and a Xe lamp of 240 mW cm^−2^. In addition, the electron spin resonance (ESR, JES-FA200) was performed for the photoexcited radicals of obtained composites. The radicals trapped with 5,5-dimethyl-l-pyrrolineN-oxide (DMPO) were carried out in water for hydroxyl radical (•O_2_^−^) and methanol for superoxide radical (•O_2_^−^) in visible light region.

### 2.3. Photocatalytic Activity

CIS/Mg(OH)_2_ composites were performed for the visible light activated photocatalytic degradation of TCH using a Xe lamp of 300 W (600 mW cm^−2^) equipped with a cut-off wavelength of 420 nm as the light source. In a typical process, 50 mg obtained bulks were dispersed into a 100 mL TCH solution and then stirred at room temperature in dark for 2.0 h to reach the adsorption-desorption equilibrium. After irradiated at an interval time, 5 mL TCH solution was sampled, filtered, and analyzed using an Agilent 1100 with a 5μm, 4.6 × 250 mm Venusil HILIC column and an ultraviolet detector of 356 nm. The Effects of CuInS_2_ content, inorganic ions, pH value and quenching agents on the photocatalytic activity of CIS/Mg(OH)_2_ were also investigated according to the above process. The intermediate products of TCH were detected by an UPLC-MS system (Waters UPLC Acquity, Quattro Premier XE).

## 3. Results

Figure S1 shows the XRD patterns of Mg(OH)_2_ and CIS/Mg(OH)_2_. The typical peaks at 18.5°, 32.7°, 37.9°, 51.2°, 58.5°, 62.0°, 68.0°, and 72.0° are assigned to the (001), (100), (101), (102), (110), (111), (103), and (201) facets of hexagonal Mg(OH)_2_ (JCPDS, 07-0239), respectively [38,39]. The diffraction peaks belonging to CuInS_2_ were not observed in XRD patterns of CIS/Mg(OH)_2_ due to its low content, low crystallinity and high dispersion [30]. In contrast with pure Mg(OH)_2_, the peak positions of Mg(OH)_2_ phases are shifted to the higher values by the introduction of CuInS_2_. 

**Figure 1:**
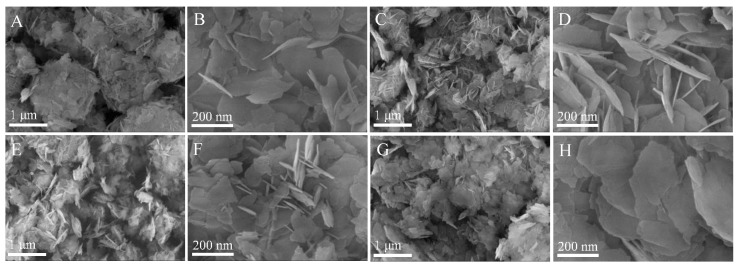
Scanning electron microscopy (SEM) images of 1-CIS/Mg(OH)_2_ (**A**,**B**), 2-CIS/Mg(OH)_2_ (**C**,**D**), 3-CIS/Mg(OH)_2_ (**E**,**F**), and 4-CIS/Mg(OH)_2_ (**G**,**H**).

**Figure 2:**
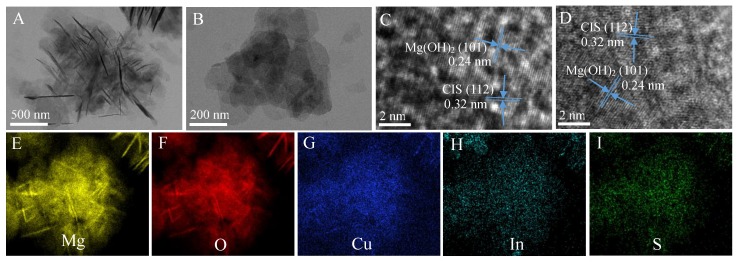
Transmission electron microscopy (TEM) images (**A**,**B**), high resolution transmission electron microscope (HRTEM) images (**C**,**D**), and elemental mapping images (**E**–**I**) of 2-CIS/Mg(OH)_2_.

SEM images (Figure S2) reveal that pure Mg(OH)_2_ is irregular flake-like structure with an average thickness of around 20 nm. The introduction of CuInS_2_ induce to the serious agglomeration degree of CIS/Mg(OH)_2_ in comparison to pure Mg(OH)_2_, as shown in Figure 1. With the increasing CuInS_2_ content, the agglomeration degree of CIS/Mg(OH)_2_ composites decreases, while their average thickness of nanosheets increases. In contrast with the sphere-like clusters of 1-CIS/Mg(OH)_2_ with a nominal CuInS_2_ content of 3.0 wt. % (Figure 1A,B), 2-CIS/Mg(OH)_2_ with a nominal CuInS_2_ content of 5.0 wt. % (Figure 1C,D) becomes weaker agglomeration, which are further confirmed by TEM images (Figure 2A,B). It’s noticed that CuInS_2_ nanoparticles of around 1.0 nm (Figure 2C,D) are well distributed in Mg(OH)_2_ nanosheets. The pacing distances of typical lattices of Mg(OH)_2_ (101) and CuInS_2_ (112) planes are 0.24 nm and 0.32 nm, respectively [32,39]. Based on the TEM image of 2-CIS/Mg(OH)_2_ (Figure S3), elemental mapping images (Figure 2E–I) also indicate that there are Mg, O, Cu, In, and S elements exist in 2-CIS/Mg(OH)_2_, meaning the successful synthesis of CuInS_2_/Mg(OH)_2_ composites. H element was not detected by elemental mapping. The N_2_ adsorption-desorption curves of obtained CIS/Mg(OH)_2_ (Figure S4) are the type IV isotherms, meaning the formation of mesopores and/or macropores structures. As listed in Table S1, the introduction of CuInS_2_ on the surface and pore structure of Mg(OH)_2_ induces to the decrease in specific surface area and pore volume.

**Figure 3:**
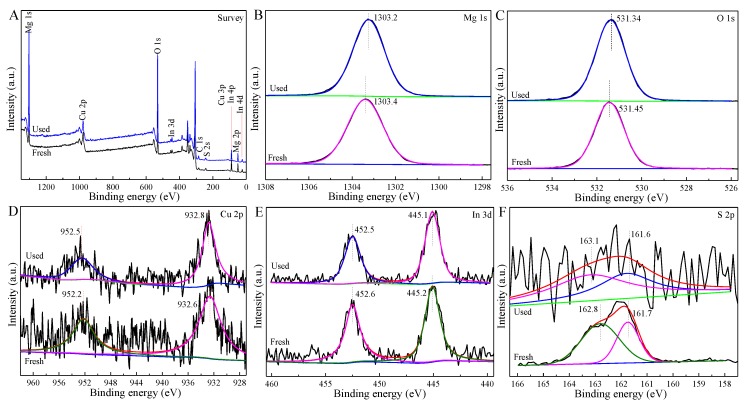
Survey (**A**), Mg 1s (**B**), O 1s (**C**), Cu 2p (**D**), In 3d (**E**), and S 2p (**F**) X-ray photoelectron spectroscopy (XPS) spectra of fresh and used 2-CIS/Mg(OH)_2_.

**Figure 4:**
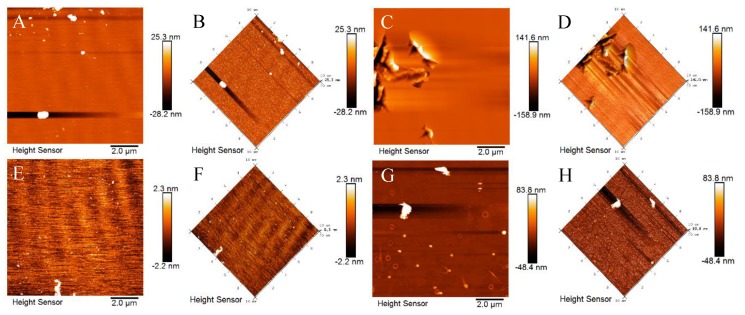
2D and 3D atomic force microscope (AFM) images of fresh (**A**,**B**), adsorbed (**C**,**D**), photocatalytic (**E**,**F**), and used 2-CIS/Mg(OH)_2_ (**G**,**H**).

As shown in Figure 3, the Mg, Cu, O, In, and S elements exist in the survey XPS spectra of fresh and used 2-CIS/Mg(OH)_2_, of which the used sample was collected after five cycle times for the visible-light driven photodegradation of TCH under the same conditions. The long-term irradiation induces to the difference in XPS spectra (Figure 3B–F) and metallic contents (Table S1) of fresh and used 2-CIS/Mg(OH)_2_. Compared with fresh sample (1303.4 eV), Mg 1s peak of used 2-CIS/Mg(OH)_2_ (Figure 4B) shifts to a lower binding energy (1303.2 eV). O 1s peaks belonging to lattice oxygen (Figure 4C) of fresh and used 2-CIS/Mg(OH)_2_ locate at 531.45 and 531.34 eV, respectively. As shown in Figure 3D, the two splitting peaks indexing to Cu 2p_1/2_ and Cu 2p_3/2_ respectively appear at 952.2 eV and 932.6 eV in fresh sample, while these peaks of used sample migrate to higher binding energies (952.5 and 932.8 eV). In contrast with fresh sample (452.6 and 445.2 eV), the peaks of In 3d_3/2_ and In 3d_5/2_ in used sample (Figure 3E) shift to the lower binding energies (452.5 and 445.1 eV). The splitting peaks of fresh 2-CIS/Mg(OH)_2_ at 162.8 161.7 eV are respectively indexed to S 2p_1/2_ and S 2p_3/2_, while these peaks of used sample respectively immigrate to 163.1 and 161.6 eV (Figure 3F).

Surface roughness of 2-CIS/Mg(OH)_2_ in different stages of photocatalytic system was observed by AFM, including the fresh, adsorbed with TCH in dark after 2.0 h, visible-light driven photocatalytic reaction after 30 min (photocatalytic) and used ones after visible-light irradiation time of 60 min (Figure 4). These adsorbed, photocatalytic, and used samples were obtained from the adsorption–photocatalysis process and not further washed with deionized water. The parameters such as average roughness (R_a_), root mean square roughness (R_q_), and surface skewness (S_sk_) obtained by Nanoscope analysis software (Table S2) were employed to describe the surface roughness of 2-CIS/Mg(OH)_2_ in the sorption-photocatalysis process for the TCH removal [48]. Due to the interface dependence of sorption-photocatalytic reaction, high surface roughness values are likely to facilitate the adsorption and photocatalysis processes, while the excessively high surface roughness induces to the mechanical and structural collapse [49]. Due to the efficient adsorption capacity and thin interpenetration layer of TCH molecules on the active sites, the R_a_ (44.2 nm), R_q_ (58.7 nm), and S_sk_ (0.45) values of this sample after adsorbed for 2.0 h at room temperature are higher than those values (8.9 nm, 15.40 nm, and 2.80) of fresh 2-CIS/Mg(OH)_2_. However, those parameters of surface roughness decrease with the increasing reaction time during the photocatalysis process, meaning the efficient conversion of TCH into small molecules and the rapid diffusion of small molecules from the bulks surface to aqueous solution. The R_a_, R_q_, and S_sk_ values of 2-CIS/Mg(OH)_2_ after irradiated 30 min are respectively 1.77 nm, 3.09 nm, and 3.06, which are higher those values (1.20 nm, 1.62 nm, and 1.00) of sample after irradiated 60 min. The reduced surface roughness of 2-CIS/Mg(OH)_2_ under long-time visible light irradiation may be ascribed to the inferior diffusion of small molecules from the active sites and photo-corrosion.

**Figure 5:**
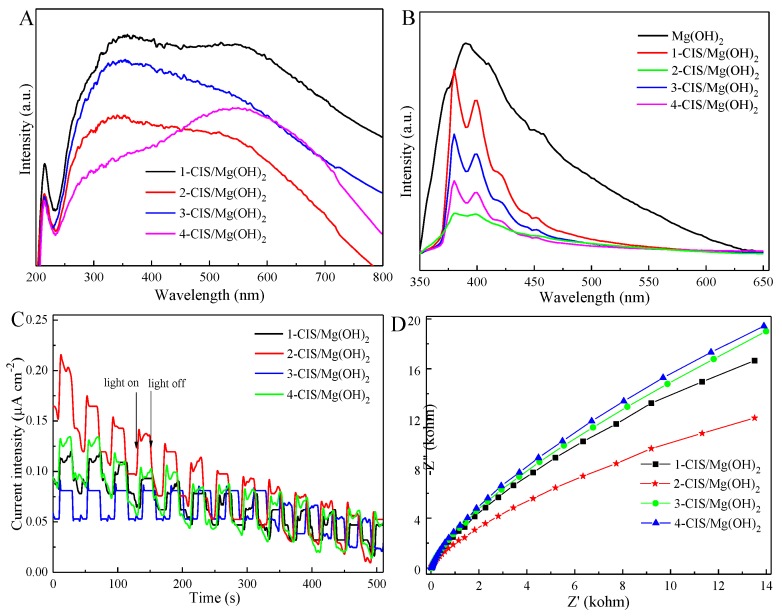
UV–Vis absorption spectra (**A**), Photoluminescence (PL) spectra (**B**), photocurrent-time response (**C**) and electrochemical impedance spectroscopy (EIS) Nynquist plots (**D**) of CIS/Mg(OH)_2_.

These CIS/Mg(OH)_2_ samples exhibit two absorption wavelengths of 200~250 nm and 250~800 nm, as shown in Figure 5A. The introduction of CuInS_2_ into Mg(OH)_2_ nanosheets can enhance the visible-light adsorption ability due to the combined effect between n-type Mg(OH)_2_ and p-type CuInS_2_ [39]. Among these composites, 2-CIS/Mg(OH)_2_ presents the best visible-light harvesting capacity. The adequate coupling of p–n semiconductors is favorable for the charge transfer and the charge carrier’s separation [50,51]. It’s noticed that PL intensity of CIS/Mg(OH)_2_ is to some extent weaker than that of Mg(OH)_2_ nanosheets, especially 2-CIS/Mg(OH)_2_ (Figure 5B). Visible light activated photocurrent responses (Figure 5C) indicates that the photocurrent intensity of 2-CIS/Mg(OH)_2_ is higher than that of X-CIS/Mg(OH)_2_ (X = 1, 3, and 4). In addition, small arc radius of electrochemical impedance is also obtained by 2-CIS/Mg(OH)_2_ (Figure 5D). It’s further indicated that the CuInS_2_ can enhance the charge transfer and restrain the recombination of photoinduced electron-hole pairs of Mg(OH)_2_ via the efficient junction interface, strengthening the photocatalytic activity.

**Figure 6:**
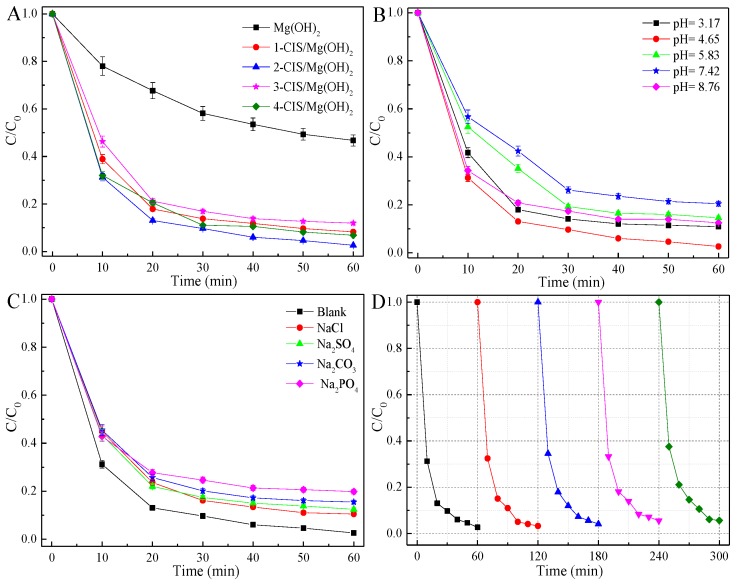
Effect of CuInS_2_ content (**A**), pH value (**B**) and inorganic ions (**C**) on the photocatalytic activity of CIS/Mg(OH)_2_ composites and the photocatalytic stability (**D**) of 2-CIS/Mg(OH)_2_.

CIS/Mg(OH)_2_ composites were performed for the photocatalytic degradation of TCH in the visible light region. The texture structure and naked active sites greatly affect the adsorption capacity and photocatalytic activity [33,51,52]. In contrasted with pure Mg(OH)_2_ nanosheets, CuInS_2_ nanoparticles loaded onto Mg(OH)_2_ induces to the inferior adsorption capacity while it enhances the photocatalytic activity under the same conditions (Table S3). As shown in Figure 6A, the photocatalytic activity of CIS/Mg(OH)_2_ increases and then decreases with an increase in CuInS_2_ content, of which 2-CIS/Mg(OH)_2_ exhibits the best photocatalytic performance in TCH removal. It’s attributed to the efficient generation and transfer of charge carriers at the junction interface between CuInS_2_ nanoparticles and Mg(OH)_2_ nanosheets. However, excess of CuInS_2_ bulk loaded on the Mg(OH)_2_ serves as the recombination center of photoinduced electron and holes, leading to the inferior photocatalytic activity [28,47]. As shown in Figure 6B, the photocatalytic activity of 2-CIS/Mg(OH)_2_ increases and then decreases with the increasing pH value below 7.0, while decreases with an increase in pH value above 7.0. Hence, the optimal pH value is 4.65 for the photocatalytic performance of 2-CIS/Mg(OH)_2_ (Table S4). The acidic condition is much more suitable for the visible-light driven photodegradation of TCH in comparison with the alkaline condition. This reason is that the amount of radicals such as •O_2_^−^ and •OH are greatly related with pH value. The OH^−^ (or H^+^) ions can react with the photoexcited e^−^/h^+^ pairs to form radical species such as •O_2_^−^ and •OH, which are crucial for the photocatalytic reaction of TCH degradation [52,53]. The chain reactions for the photo-activated radicals are seriously quenched by excess of OH^−^ and H^+^ ions, leading to the inferior photocatalytic activity. In addition, inorganic ions play an important role in the visible light driven photocatalytic capacity. The addition of inorganic ions can not only affect the pH value of TCH solution, but also scavenge the radical species in the photocatalytic system [51,54]. It’s noticed that the inorganic ions such as NaCl, Na_2_SO_4_, Na_2_CO_3_, and Na_3_PO_4_ restrain the photocatalytic capacity of 2-CIS/Mg(OH)_2_ for TCH removal in visible light region (Figure 6C and Table S5). 2-CIS/Mg(OH)_2_ slightly deactivates for the visible light driven degradation of TCH after five cycle times (Figure 6D), indicating the excellent durability and recyclability of 2-CIS/Mg(OH)_2_. This was further confirmed by XRD, SEM images, and XPS. Compared with fresh 2-CIS/Mg(OH)_2_, the XRD pattern (Figure S5) and morphology structure (Figure S6) of used sample are not changed. However, the surface composition (Figure 3) is changed due to the long-time irradiation in visible light region.

**Figure 7:**
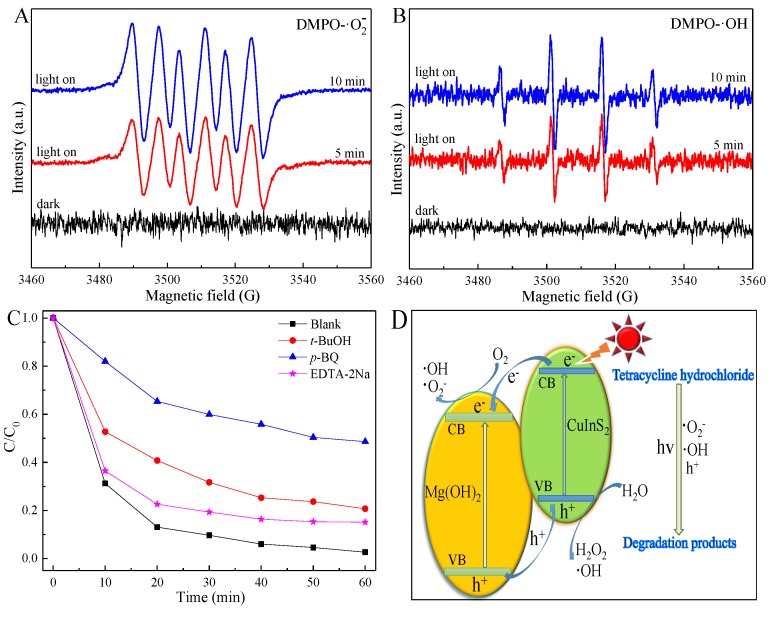
Electron spin resonance (ESR) spectra of DMPO-·O_2_^−^ in methanol (**A**) and DMPO-·OH in aqueous (**B**), quenching test (**C**), and photocatalytic mechanism (**D**) of 2-CIS/Mg(OH)_2_.

The radical species such as •O_2_^−^ and •OH were evaluated by ESR. The ESR signals intensities of DMPO-•O_2_^−^ in methanol (Figure 7A) are stronger than those of DMPO-·OH in aqueous (Figure 7B), which further strengthen with an increase in the irradiation time. It’s suggested that •O_2_^−^ radical plays a crucial role in the photocatalytic reaction of 2-CIS/Mg(OH)_2_, which is further evaluated by the quenching test. The addition of scavengers (0.1 mmol) such as tert-butyl alcohol (*t*-BuOH), p-benzoquinone (*p*-BQ) and ethylenediamine tetraacetic acid disodium salt (EDTA-2Na) into the photocatalytic system can restrain the photocatalytic activity of 2-CIS/Mg(OH)_2_ (Figure 7C). Compared with EDTA-2Na and *t*-BuOH, the photocatalytic activity of 2-CIS/Mg(OH)_2_ is greatly suppressed by the addition of *p*-BQ. It’s suggested that •O_2_^−^ radical is the vital radical for the TCH removal. 

The possible photocatalytic mechanism of 2-CIS/Mg(OH)_2_ is proposed in Figure 7D. In this heterojunction structure, the photoexcited electrons generated from the valence band (VB) of CuInS_2_ are transferred from the conduction band (CB) of CuInS_2_ to the CB of Mg(OH)_2_, meanwhile the h^+^ radicals formed from VB of Mg(OH)_2_ are migrated to VB of CuInS_2_. This facilitates the repaid transfer and separation of photoexcited e^−^ and H^+^, leading to the efficient photocatalytic activity. Dissolved O_2_ molecules can react with e^−^ to form •O_2_^−^ and •OH radicals, and H_2_O molecules can react with h^+^ to generate •OH radical. These radicals can efficiently attacked TCH molecules into small molecules through the demethylation, hydroxylation, deamination, N–C bond cleavage, loss of NH_3_ and H_2_O (Figures S7 and S8), which can be further strengthened with the increasing irradiation time to generate non-toxic molecules [8,55]. The enhanced photocatalytic capacity of 2-CIS/Mg(OH)_2_ composite is achieved for the visible light driven degradation of TCH compared with the previous works, as listed in Table S6. Especially, TiO_2_ is not excited in visible light region but in UV-light region, and its photocatalytic activity is greatly affected by its microstructure, crystalline phases, and naked lattice planes [56,57,58,59,60]. 2-CIS/Mg(OH)_2_ can be considered as an efficient photocatalyst for the visible-light driven treatment of wastewaters such textile and antibiotics effluents in industrial application.

## 4. Conclusions

CIS/Mg(OH)_2_ nanosheets exhibit the better photocatalytic activity in comparison to Mg(OH)_2_ for the visible light driven degradation of TCH. The combined effect between CuInS_2_ and Mg(OH)_2_ is likely to enhance the photocatalytic activity. The surface roughness of 2-CIS/Mg(OH)_2_ is changed in adsorption-photocatalytic system. ESR results indicate that •O_2_^−^ radical as well as •OH is the main radical for the enhanced photocatalytic activity of 2-CIS/Mg(OH)_2_. Among these obtained composites, 2-CIS/Mg(OH)_2_ with the nominal CuInS_2_ content 5.0 wt. % presents the best photocatalytic activity and stability after five cycle times under the same conditions.

## Supplementary Materials

The following are available online at https://www.mdpi.com/2079-4991/9/11/1567/s1, Figure S1: XRD patterns of Mg(OH)_2_ and CIS/Mg(OH)_2_, Figure S2: SEM images of Mg(OH)_2_, Figure S3: TEM images of 2-CIS/Mg(OH)_2_, Figure S4: N_2_ adsorption-desorption curves of CIS/Mg(OH)_2_ with varying CuInS_2_ content, Figure S5: XRD patterns of fresh and used 2-CIS/Mg(OH)_2_, Figure S6: SEM images of used 2-CIS/Mg(OH)_2_, Figure S7: HPLC/MS spectrum of TCH solution under visible-light irradiation of 30 min over 2-CIS/Mg(OH)_2_, Figure S8: HPLC/MS spectrum of TCH solution under visible-light irradiation of 60 min over 2-CIS/Mg(OH)_2_; Table S1: Texture parameters of CIS/Mg(OH)_2_-based samples, Table S2: The parameters of Surface roughness of 2-CIS/Mg(OH)_2_, Table S3: The adsorption-photocatalysis capacities of CIS/Mg(OH)_2_ samples, Table S4: Effect of pH value on the adsorption-photocatalysis capacities of 2-CIS/Mg(OH)_2_, Table S5: Effect of inorganic ions on the adsorption-photocatalysis capacities of 2-CIS/Mg(OH)_2_, Table S6: Comparison of photocatalytic performance for removal of organic pollutants.
