# Supplementary material for: CuInS2/Mg(OH)2 Nanosheets for the Enhanced Visible-Light Photocatalytic Degradation of Tetracycline
Source: Nanomaterials (Basel). 2019 Nov 5;9(11):1567. doi: 10.3390/nano9111567 (PMC6915496; doi:10.3390/nano9111567)
Supplement: Supplementary file 1 [file nanomaterials-09-01567-s001.pdf]

Supported Information

$CuInS_2/Mg(OH)_2$ nanosheets for the enhanced visible-light photocatalytic degradation of tetracycline

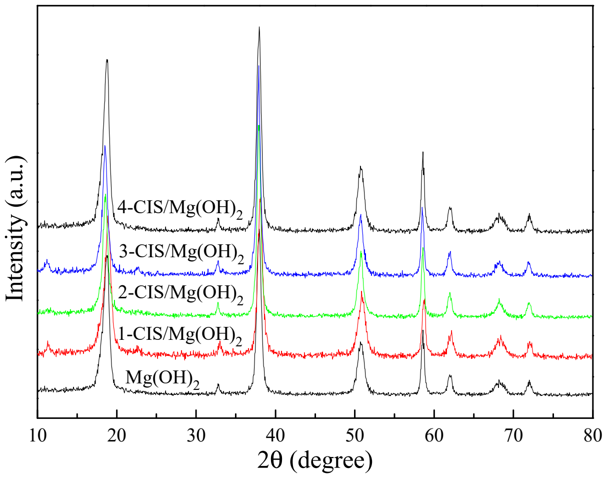

Figure S1. XRD patterns of $Mg(OH)_2$ and CIS/$Mg(OH)_2$.

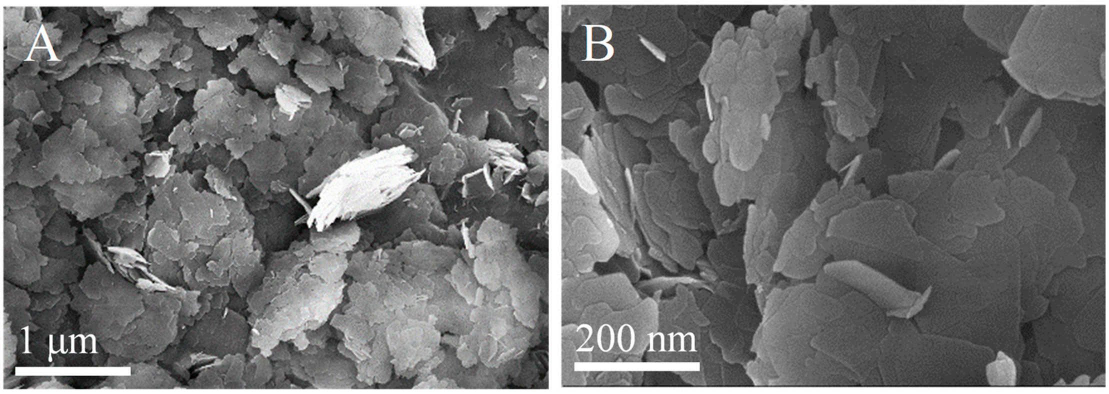

Figure S2. SEM images of $Mg(OH)_2$.

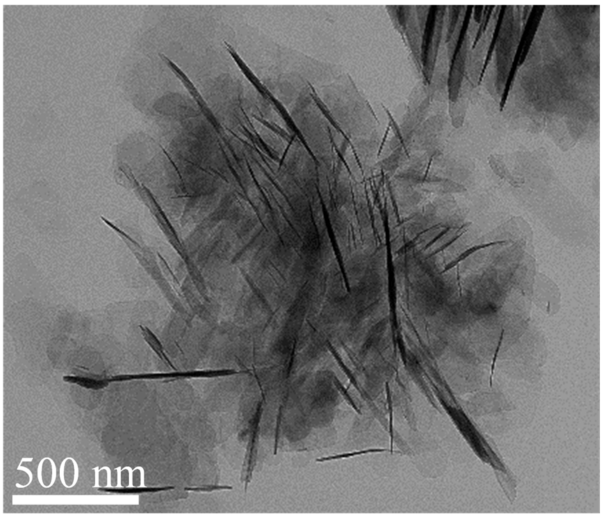

Figure S3. TEM images of 2-CIS/Mg(OH)$_2$.

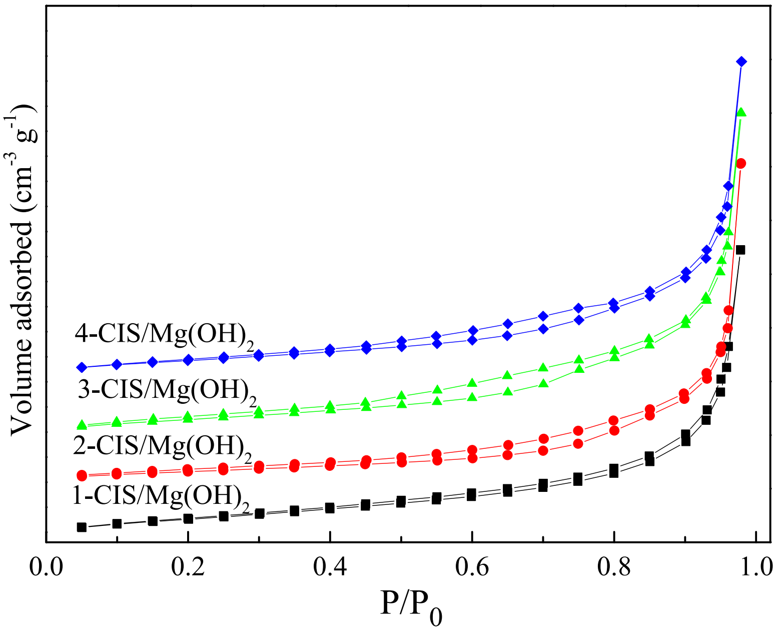

Figure S4. N_2 adsorption-desorption curves of CIS/Mg(OH)$_2$ with varying $CuInS_2$ content.

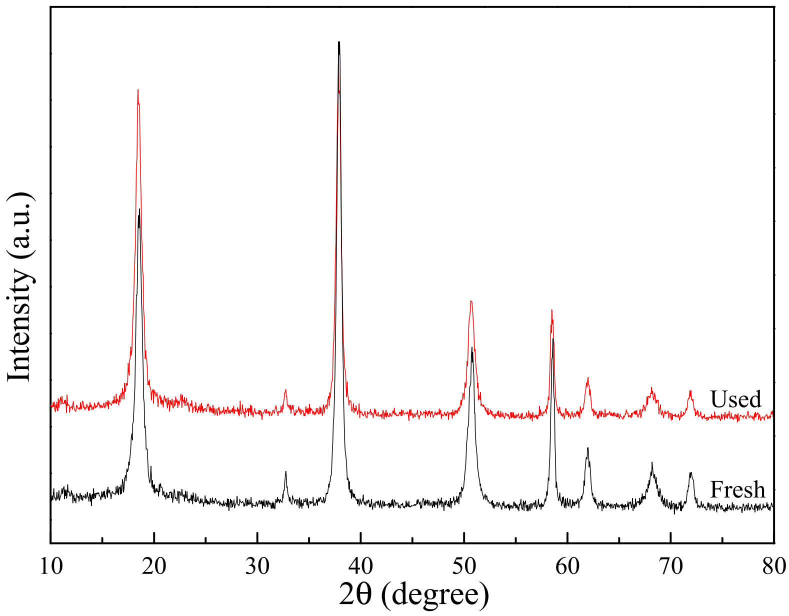

Figure S5. XRD patterns of fresh and used 2-CIS/$Mg(OH)_2$.

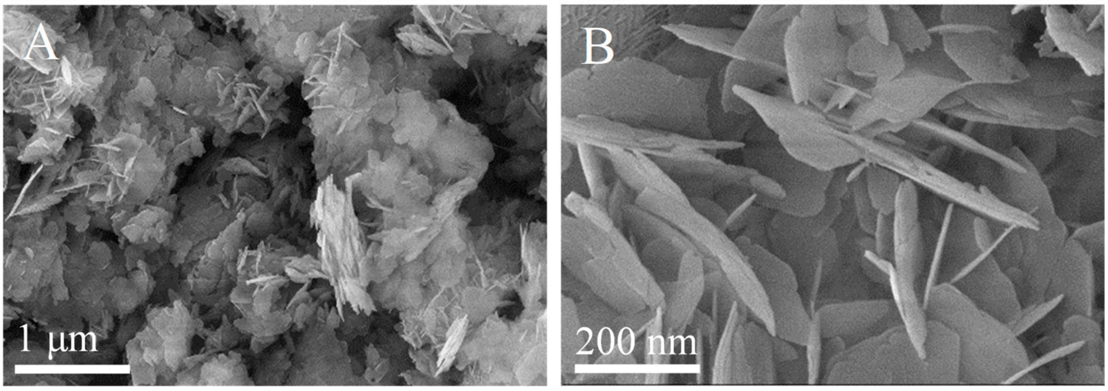

Figure S6. SEM images of used 2-CIS/$Mg(OH)_2$.

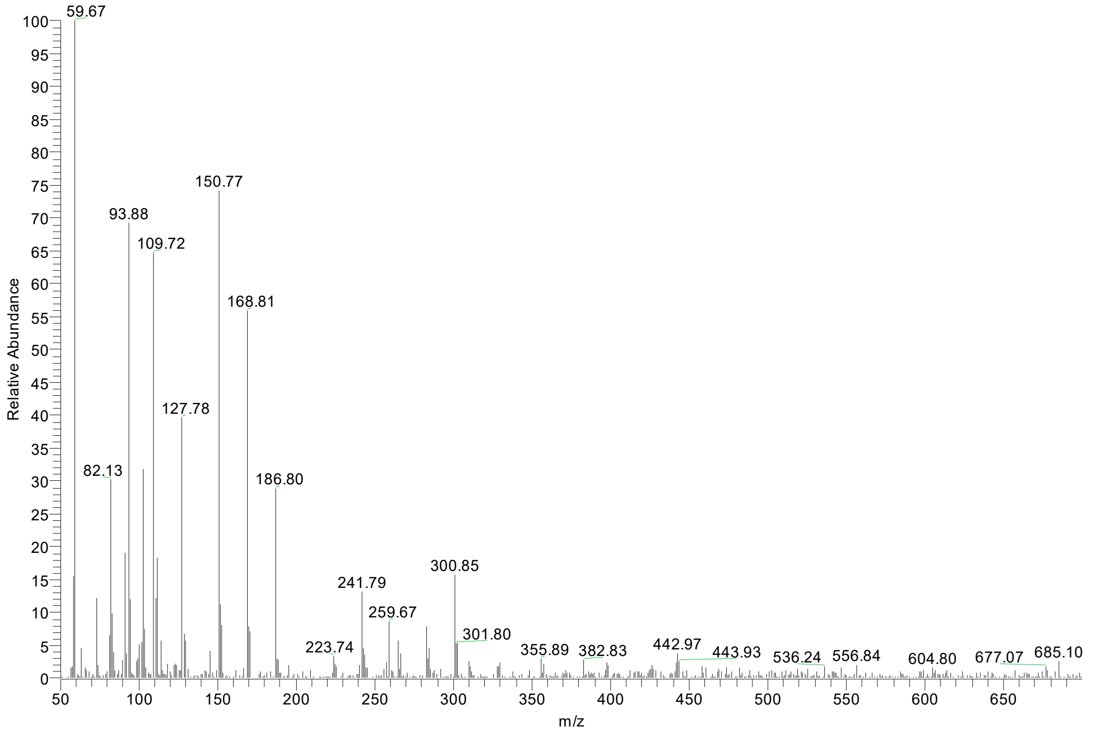

Figure S7. HPLC/MS spectrum of TCH solution under visible-light irradiation of 30 min over 2-CIS/$Mg(OH)_2$.

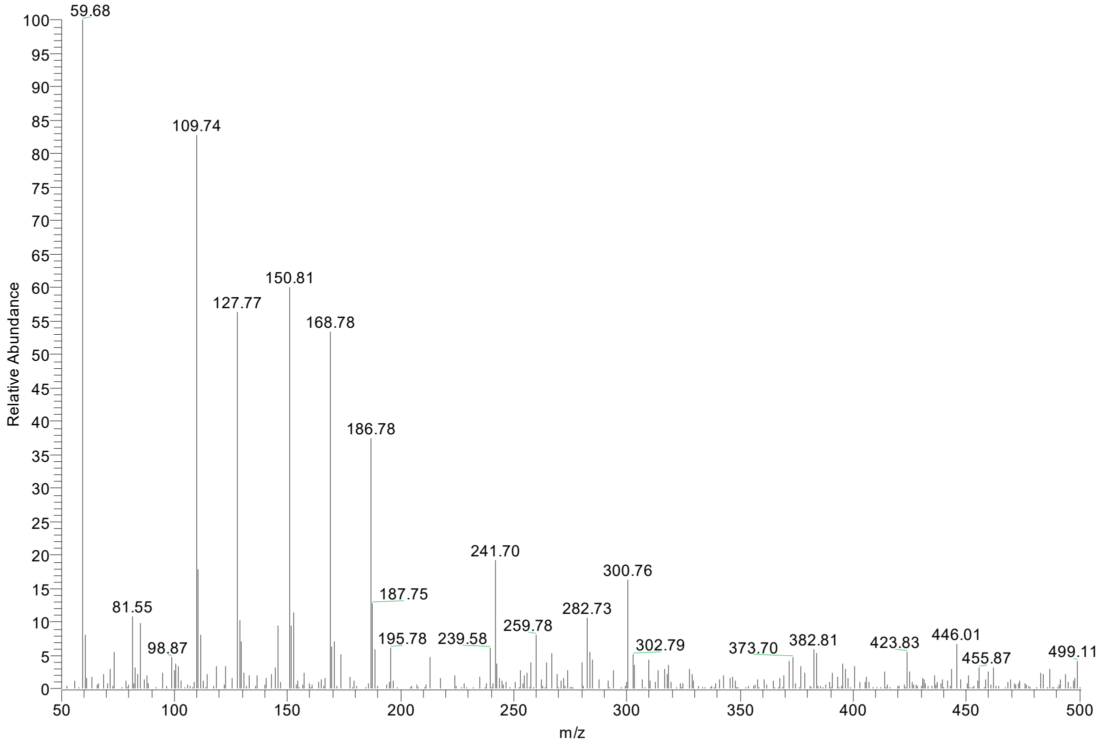

Figure S8. HPLC/MS spectrum of TCH solution under visible-light irradiation of 60 min over 2-CIS/$Mg(OH)_2$.

Table S1. Texture parameters of CIS/$Mg(OH)_2$-based samples.

Samples	BET surface area (m^2 g^{-1})	Pore volume (cm^3 g^{-1})	Pore diameter (nm)	Metallic content (%) [a]		
				Mg	Cu	In
1-CIS/$Mg(OH)_2$	46.7	1.25	3.06	40.59	0.51	0.92
2-CIS/$Mg(OH)_2$	38.8	1.09	3.21	39.15	1.04	1.73
3-CIS/$Mg(OH)_2$	33.4	0.98	3.64	38.86	1.67	3.10
4-CIS/$Mg(OH)_2$	30.5	0.91	3.52	37.69	1.83	3.41
Used 2-CIS/$Mg(OH)_2$[c]	/	/	/	38.52	0.98	1.75

[a] The contents of metallic elements was measured by ICP-OES.

[b] The atomic contents of metallic elements was measured by XPS.

[c] The used composite was not detected by N_2-sorption for the BET, pore volume and pore diameter.

Table S2. The parameters of Surface roughness of 2-CIS/$Mg(OH)_2$.

Samples	R_a (nm) [a]	R_q (nm) [b]	S_{sk} [c]
Fresh	8.90	15.40	2.80
Adsorption [d]	44.2	58.7	0.45
Photocatalysis [e]	1.77	3.09	3.06
Used [f]	1.20	1.62	1.00

[a] Ra was the average roughness, nm.

[b] Rq was the root mean square roughness, nm.

[c] S_{sk} was the surface skewness.

[d] The adsorption time is 2.0 h.

[e] The photocatalytic time is 30 min.

[f] The photocatalytic time is 60 min.

Table S3. The adsorption-photocatalysis capacities of CIS/$Mg(OH)_2$ samples.

Samples	Adsorption		photocatalysis		Removal (%)
	C_0 (mg L^{-1})	Efficiency [a] (%)	C (mg L^{-1})	Efficiency [b] (%)	
$Mg(OH)_2$	26.75	46.50	12.51	53.23	74.98
1-CIS/$Mg(OH)_2$	38.02	23.96	3.14	91.74	93.71
2-CIS/$Mg(OH)_2$	37.54	24.92	0.03	99.92	99.95
3-CIS/$Mg(OH)_2$	39.23	21.54	4.68	88.07	90.64
4-CIS/$Mg(OH)_2$	37.70	24.60	2.58	93.16	94.83

[a] Adsorption efficiency = $(50-C_0)/50*100\%$. [b] Photocatalysis efficiency = $(C_0-C)/C_0*100\%$.

Conditions: TCH concentration of 50 mg L^{-1}, Photocatalyst bulks of 50 mg, TCH solution volume of 100 mL, Light power intensity of 600 mW cm^{-2}, pH value of 4.65 and Adsorption time of 2.0 h.

Table S4. Effect of pH value on the adsorption-photocatalysis capacities of 2-CIS/$Mg(OH)_2$.

pH	Adsorption		photocatalysis		Removal (%)
	C_0 (mg L^{-1})	Efficiency [a] (%)	C (mg L^{-1})	Efficiency [b] (%)	
3.17	40.75	18.50	4.46	89.06	91.09
4.65	37.54	24.92	0.03	99.92	99.95
5.83	34.63	30.74	5.06	85.39	89.89
7.42	27.18	45.64	5.56	79.54	88.87
8.76	37.28	25.44	4.66	87.50	90.67

[a] Adsorption efficiency = $(50-C_0)/50\times100\%$. [b] Photocatalysis efficiency = $(C_0-C)/C_0\times100\%$.

TCH concentration of 50 mg L^{-1}, Photocatalyst bulks of 50 mg, TCH solution volume of 100 mL, Light power intensity of 600 mW cm^{-2} and Adsorption time of 2.0 h.

Table S5. Effect of inorganic ions on the adsorption-photocatalysis capacities of 2-CIS/$Mg(OH)_2$.

Samples	Adsorption		photocatalysis		Removal (%)
	C_0 (mg L^{-1})	Efficiency [a] (%)	C (mg L^{-1})	Efficiency [b] (%)	
Blank	37.54	24.92	0.03	99.92	99.95
NaCl	37.69	24.62	3.95	89.52	92.09
Na_2SO_4	38.42	23.16	4.79	87.53	90.43
Na_2CO_3	42.17	15.66	6.53	84.52	86.94
Na_3PO_4	46.47	7.06	9.22	80.16	81.55

[a] Adsorption efficiency = $(50-C_0)/50\times100\%$. [b] Photocatalysis efficiency = $(C_0-C)/C_0\times100\%$.

TCH concentration of 50 mg L^{-1}, Photocatalyst bulks of 50 mg, TCH solution volume of 100 mL, Visible-light power intensity of 600 mW cm^{-2}, pH value of 4.65 and Adsorption time of 2.0 h.

Table S6. Comparison of photocatalytic performance for removal of organic pollutants.

Samples	Light source		Organic pollutants			η [b] (%)	Ref.
	Type [a]	P (W)	Type	C(mg L^{-1})	T(min)		
Ag/TiO_2	Xe	300	Tetracycline	30	600	90.0	8
MgO@N-C	Xe	300	Tetracycline	400	70	92.20	15
Fe_2O_3@TiO_2 composites	Xe	300	Tetracycline	50	90	100	16
$CuInS_2$/g-C_3N_4	Xe	300	Tetracycline	20	60	83.70	31
TiO_2	Hg	9	Tetracycline	30	90	78.2	55
Core-shell TiO_2@α-Fe_2O_3	Xe	300	Methyl orange	10	16	96.6	56
Core-shell Fe_2O_3/TiO_2	Hg	450	Paracetamol	50	90	87.8	57
Flower–like α-Fe_2O_3@TiO_2	Hg	300	Acid orange 7	10	90	98.6	58
α-Fe_2O_3@TiO_2 nanorods	Hg	300	Rhodamine B	100	120	100	59
TiO_2 P-25	Hg	125	Tetracycline	40	120	100	60
2-CIS/$Mg(OH)_2$	Xe	300	Tetracycline	50	60	99.95	Present

[a] Hg lamp were the UV-light sources, and Xe lamp was the visible-light source.

[b] η was the total removal efficiency of organic pollutant.
